# Standardized protocol for plasticity assessment in the aging mouse neocortex using choline-chloride perfusion

**DOI:** 10.3389/fnagi.2026.1764324

**Published:** 2026-03-12

**Authors:** Pia Kruse, Charlotte Schob, Kerstin Schwabe, Maximilian Lenz

**Affiliations:** 1 Hannover Medical School, Institute of Neuroanatomy and Cell Biology, Hannover, Germany; 2 Center for Systems Neuroscience, Hannover, Germany; 3 Department of Neurosurgery, Hannover Medical School, Hannover, Germany

**Keywords:** acute slicing, choline-chloride, cLTP, forskolin, perfusion, synaptic plasticity

## Abstract

Age-related changes in synaptic function are central to the progression of brain pathologies, including neurodegenerative diseases, underscoring the need for experimental approaches that capture neuronal properties across the lifespan. However, obtaining high-quality tissue preparations from aged animals that permit combined structural and functional analyses of individual neurons is challenging due to increased tissue vulnerability. Here, we present a standardized protocol for acute brain slice preparation using transcardial choline-chloride perfusion to reliably obtain intact cortical slices from mice at different ages (young mice: 7–10 weeks old; aged mice: 9–11 months old). Using the medial prefrontal cortex (mPFC) as an example, we show that cortical lamination and subcellular synaptic structure are preserved in supragranular (layer 2/3) pyramidal neurons. Subsequently, we examined spontaneous excitatory synaptic transmission by whole-cell patch-clamp recordings. We demonstrated that forskolin-induced chemical long-term potentiation (cLTP) can be reliably induced and measured in both young and aged slices, revealing age-related differences in the expression of synaptic plasticity. This protocol provides a reproducible framework for investigating synaptic transmission and plasticity in the aging cortex and is broadly applicable to studies of age-related brain disorders.

## Introduction

Aging is the predominant risk factor for neurodegenerative diseases. Profound changes in neuronal networks in the aging brain can be observed, ultimately leading to a decline in cognitive, sensory, or motor performance (Wen et al., 2025). To enable learning, memory and higher cognitive functions, previous work has identified the ability of neurons and their synapses to adapt to internal or external stimuli at the structural, functional, and molecular level as a fundamental prerequisite (Morris et al., 1986; Turrigiano et al., 1998). Consequently, the functional and structural assessment of neurons and their potential to adjust their synaptic properties, particularly in the aging brain, is essential to gain deeper insights into the mechanisms underlying neurological pathologies and age-related cognitive decline.

Over the past decades, the utilization of *ex vivo* acute brain slice preparations for electrophysiological recordings has been established to study functional properties of neurons (Campagnola et al., 2022; Whitebirch et al., 2022). While many experiments have been conducted in young adult mice (~8–10 weeks), the systematic investigation of neuronal networks and synaptic transmission in the aging brain (> 8 months) remains sparse (Ting et al., 2014). Due to age-related changes in brain tissue composition, such as a higher degree of myelination leading to increased stiffness (Chuang et al., 2023) as well as a higher vulnerability to hypoxia and osmotic stress (Wang et al., 2018; Djurisic, 2020; Kolenicova et al., 2020), the preparation of acute slices from aged animals that are suitable for single-cell investigation poses a major experimental challenge. Various experimental protocols have been suggested using N-methyl-D-glucamine- (NMDG-), sucrose-, or choline-based solutions to reduce Na^+^-induced excitotoxicity during the slicing process (Ting et al., 2018; Saunders et al., 2015; McIntyre et al., 2002). While NMDG- and choline-based solutions appear to be superior to sucrose in aged animals (Ting et al., 2018; Perumal and Sah, 2022; Popescu and Pare, 2011), the preparation of NMDG-based solutions can be time-consuming and error-prone. In contrast, choline-chloride-based approaches offer a technically straightforward alternative that can be readily implemented in standard electrophysiological procedures.

**Figure 1:**
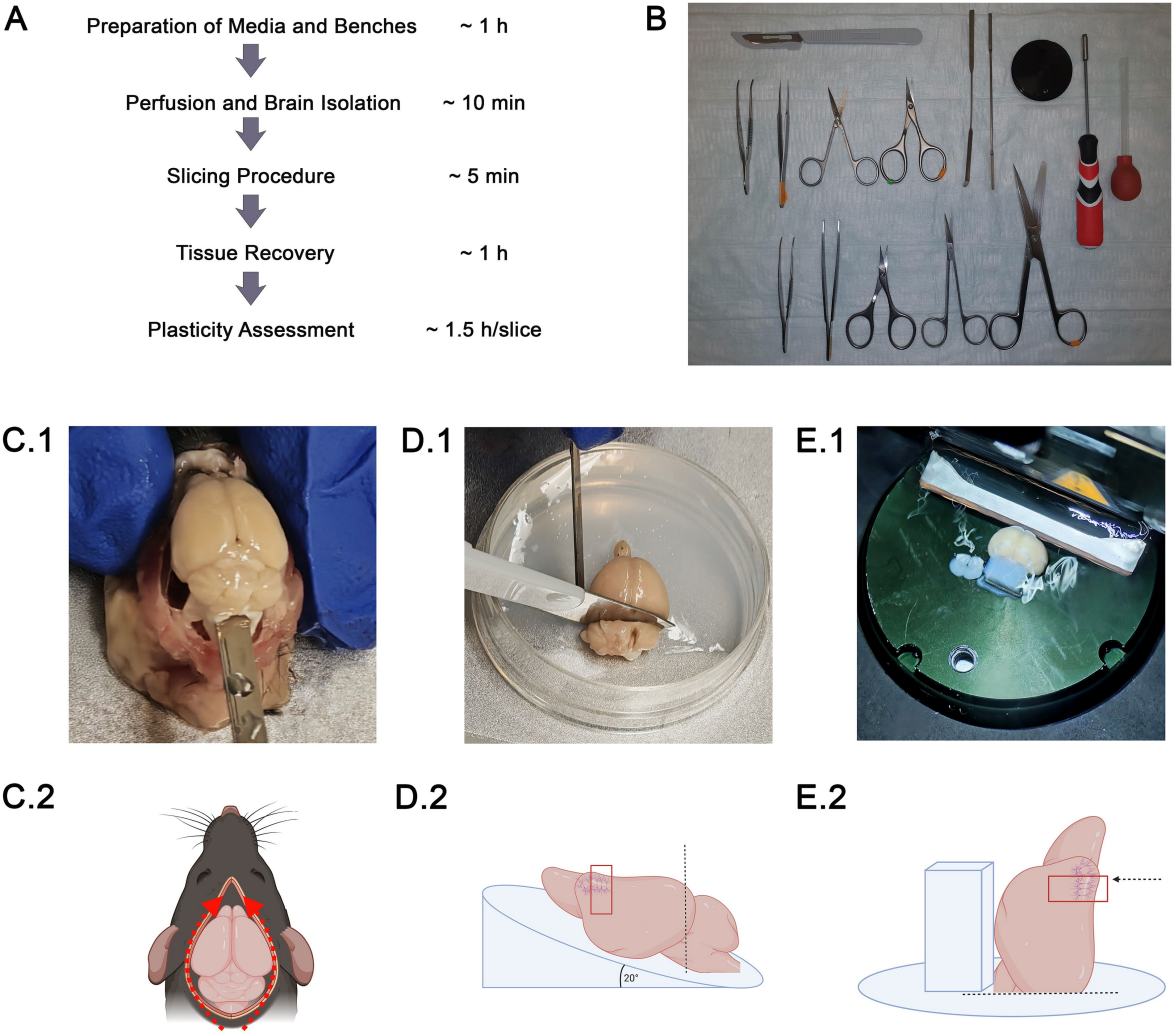
Acute slice preparations of the medial prefrontal cortex (mPFC) in young and aged animals. **(A)** Workflow of experimental procedure from preparation to plasticity assessment with approximate timing. **(B)** Image of instruments that are needed for perfusion and brain isolation. **(C)** Brain isolation with a spatula after skull is carefully opened by lateral cuts and the skullcap is removed with forceps. **(D)** The brain is placed on an agarose slope with a tilted angle of 20° for optimizing the cutting angle that generates a flat posterior surface. The angle is needed to avoid truncation of neurons in the region of interest. **(E)** The brain is glued on the flat posterior surface with an agarose block on the ventral side for additional stabilization. Then, the slicing procedure can be started using a vibratome.

Here, we report an improved workflow using transcardial choline-chloride perfusion to obtain acute neocortical slices from aged mice that are suitable for whole-cell patch-clamp recordings and the assessment of synaptic plasticity with single-cell resolution (Figure 1). We transparently report and refine a step-by-step experimental workflow, including tissue handling and slicing procedures, together with the identification of critical steps and practical troubleshooting strategies for commonly observed problems, as detailed in the methodological description. Because synaptic plasticity mechanisms such as long-term potentiation (LTP) are thought to underlie learning and higher cognitive function, reliable assessment of plasticity across age is critical. This underscores the relevance of standardized approaches to study plasticity in aged cortical tissue. Using the medial prefrontal cortex (mPFC) as a use case, we demonstrate that neurons maintain their functional and structural integrity after the slicing procedure in both young and aged animals. We recorded spontaneous excitatory postsynaptic currents (sEPSCs) from supragranular pyramidal neurons (PNs) and subsequently assessed their ability to express synaptic plasticity by forskolin-induced chemical long-term potentiation (cLTP), which is a robust, pharmacologically induced form of synaptic strengthening (Otmakhov et al., 2004; Ando et al., 2010; Ma et al., 2024). Our results show that excitatory synaptic plasticity, i.e., forskolin-induced synaptic changes, is preserved in both young and aged acute neocortical slices, albeit with distinct age-dependent characteristics. Thus, our protocol provides a standardized experimental framework to investigate synaptic plasticity in aged neocortical slices and can, in principle, be adapted to other cortical and subcortical areas. The aim of this protocol is to enable reproducible and convenient assessment of fundamental changes in synaptic structure and function in the aging brain.

## Materials and equipment

### Reagents

**Table 1:** 

Chemicals/materials	Provider	Cat. number
Adenosine 5′-triphosphate magnesium salt	Sigma-Aldrich, USA	A9187
Agarose, low EEO	Sigma-Aldrich, USA	A0576
Biocytin	Sigma-Aldrich, USA	B4261
Calcium chloride solution (1 M)	Sigma-Aldrich, USA	21115
Choline-chloride	Sigma-Aldrich, USA	C7527
D-(+)-Glucose solution (45%)	Sigma-Aldrich, USA	G8769
DAKO fluorescence mounting medium	Agilent Technologies, USA	S3023
DAPI	Thermo Fisher Scientific, Germany	62248
Forskolin	Alomone Labs, Israel	F-500
Guanosine 5′-triphosphate (sodium salt hydrate)	Cayman Chemical, USA	16060
HEPES buffer solution (1 M)	Gibco, Germany	15630–056
Isoflurane CP 1 mL/ml	CP Pharma, Germany	1214
Magnesium chloride	Carl Roth GmbH & Co. KG, Germany	KK36.1
Normal Goat Serum	Gibco, Germany	16210–064
Paraformaldehyde (4%)	Sigma-Aldrich, USA	158127
Phosphate-buffered saline	Merck KGaA, Germany	L182-50
Phosphocreatine disodium salt hydrate	Sigma-Aldrich, USA	P7936
Potassium chloride	Carl Roth GmbH & Co. KG, Germany	6781.1
Potassion D-gluconate	Sigma-Aldrich, USA	G4500
Potassium hydroxide	Carl Roth GmbH & Co. KG, Germany	7986.1
Sodium chloride	J. T. Baker, USA	0278
Sodium hydrogen carbonate	Sigma-Aldrich, USA	S6014
Sodium hydrogen phosphate monohydrate	Carl Roth GmbH & Co. KG, Germany	K300.1
Sodium hydroxide solution (1 M)	Carl Roth GmbH & Co. KG, Germany	K021.1
Streptavidin, Alexa Fluor 647 Conjugate	Invitrogen, USA	S32357
Triton X-100	Sigma-Aldrich, USA	T8787-088

### Formulations

**Table 2:** 

Choline-chloride solution (perfusion and cutting solution)	
118 mM	Choline-chloride
26 mM	Sodium hydrogen carbonate
10 mM	D-(+)-Glucose
2.5 mM	Potassium chloride
1.25 mM	Sodium hydrogen phosphate
2.5 mM	Calcium chloride
7 mM	Magnesium chloride

**Table 3:** 

Artificial cerebrospinal fluid (aCSF; recovery and recording solution)	
126 mM	Sodium chloride
26 mM	Sodium hydrogen carbonate
10 mM	D-(+)-Glucose
2.5 mM	Potassium chloride
1.25 mM	Sodium hydrogen phosphate
2 mM	Calcium chloride
2 mM	Magnesium chloride

**Table 4:** 

Patch clamp internal solution	
126 mM	Potassion D-gluconate
10 mM	HEPES
10 mM	Phosphocreatine disodium salt hydrate
4 mM	Potassium chloride
4 mM	Adenosine 5′-triphosphate magnesium
0.3 mM	Guanosine 5′-triphosphate
0.2% (w/V)	Biocytin
Prepare solution on ice. pH 7.25 with Potassium hydroxide, 285 mOsm/kg	

For optimal results, deionized water should be used for the preparation of internal and external solutions. Internal solution should be aliquoted and stored at −20°. It can be used for several months. External solutions should be kept at 4° and can be used for up to 3 days.

### Equipment

**Table 5:** 

Instrument	Manufacturer	Cat. number
Disposal Scalpel No. 21 pfm	Feather Safety Razor Co., LTD, Japan	02.011.30.021
Dumont #55 Forceps	Fine Science Tools GmbH, Germany	11255-20
Graefe Forceps	Fine Science Tools GmbH, Germany	11051-10
Hex with ball with handle 2,5 × 75 mm YT1531	Yato, China	YT-1531
Omnifix Luer Solo, 10 mL	B. Braun, Germany	4616103 V
Pasteur Pipets by Labsolute	Th. Geyer GmbH & Co. KG, Germany	7691060
Pipetting aid Suction bulbs	Witeg Lebortechnik GmbH, Germany	3597001
Safety-Multifly 21G tube200mm	Sarstedt AG & Co. KG, Germany	85.1638.235
Sample Plate, n. o. 20 mm	Leica Microsystems, Germany	14048142086
Standard Surgical Scissors – Sharp/Blunt	Fine Science Tools GmbH, Germany	14001-16
Stainless Steel Micro Chattaway Spatulas, 150 mm	Fisherbrand, Germany	11543482
Student Bone Scissors	Fine Science Tools GmbH, Germany	91604-09
Student Iris Scissors	Fine Science Tools GmbH, Germany	91460-11
Sugical Standard Forceps – 1×2 Teeth	Fine Science Tools GmbH, Germany	11021-12

**Table 6:** 

Device	Manufacturer
Axon Digidata 1550B	Molecular Devices Electrophysiology, USA
Axon MultiClamp 700B Commander	Molecular Devices Electrophysiology, USA
Control panel VT1200S	Leica Microsystems, Germany
DMi 8 confocal microscope	Leica Microsystems, Germany
EverFlo Oxygen Concentrator	Philips Respironics, Germany
F250 Compact Circulation Cooler	JULABO, Germany
HeraGuard Eco Clean Bench	Thermo Fisher Scientific, Germany
Isoflurane Vaporizer	Rothacher – Medical GmbH, Switzerland
LNscope	Luigs & Neumann, Germany
ORCA-spark Digital CMOS camera	Hamamatsu Photonics, Japan
P-1000 Flaming/Brown Micropipette Puller	Sutter Instruments, USA
SM10 Remote Control Touch	Luigs & Neumann, Germany
Vibratome VT1200S	Leica Microsystems, Germany

**Table 7:** 

Software	Developer
Axon MultiClamp Commander	Molecular Devices, LLC., USA
Clampfit 11.3	Molecular Devices, LLC., USA
Clampex	Molecular Devices, LLC., USA
GraphPad Prism 10	GraphPad Software, LLC., USA
LAS X	Leica Microsystems, Germany
TOKUPIC	Hamamatsu Photonics K. K., Japan

## Methods and experimental protocol

### Ethics statement

All experimental procedures were performed in accordance with German animal welfare legislation and were approved by the responsible institutional animal welfare committee and the animal welfare officer at Hannover Medical School (AZ 2023/257). Mice were housed at the Central Animal Laboratories (ZTL, Hannover Medical School) under a 14 h light/10 h dark cycle with food and water available *ad libitum*. Experiments were designed and reported in accordance with ARRIVE guidelines. Every effort was made to minimize distress and pain for the animals. For this study, female C57BL/6 J mice were used at two age points (3 mice per age; young: 7–10 weeks old; aged: 9–11 months old).

### Overview step-by-step protocol

A short summary of the main steps and timing of the protocol is shown in Figure 1A.

**Table 8:** 

Procedure	Step	Timing
Preparation of media and benches	Preparation of external solutions (choline-chloride and aCSF) and agarose slope	30 min
	Set up vibratome and benches (for perfusion and slicing procedure)	30 min
		~ 1 h
Perfusion + Brain isolation	Anaesthesia and perfusion	~ 5 min
	Brain isolation, orientation and attachment of brain to specimen table	~ 5 min
		~ 10 min
Slicing Procedure	Slicing procedure and tissue transfer to recovery chamber	~ 5 min
		~ 5 min
Tissue recovery	Tissue recovery at 35° C	30 min
	Tissue in holding chamber at RT	> 30 min
		~ 1 h
Plasticity assessment	Electrophysiological assessment of neurons prior to forskolin treatment	~35 min
	Forskolin wash-in and wash-out	20 min
	Electrophysiological assessment of neurons after forskolin treatment	~35 min
		~ 1.5 h/slice

### Acute slice preparation

#### Preparation (~ 1 h)

1. Prepare the choline-chloride solution for transcardial perfusion and acute slice preparation (0.5 L per mouse) and the aCSF for recovery, holding, and recording (1.5 L aCSF per mouse). After preparation, solutions can be stored in the refrigerator at 4 °C for up to 3 days and should be thoroughly re-oxygenated before use.
2. Freshly prepare 1.7% agarose (w/v in aCSF) and use a 35 mm Petri dish to pour in the liquid agarose while tilting the dish at approximately 20° to generate an angled agarose surface, which is essential for adjusting the mPFC cutting angle. The angle of the agarose surface might be adjusted to the target brain region in order to avoid truncation of dendrites.
3. Set up the vibratome: clean the cutting chamber and blade holder with 70% ethanol (v/v). Clean the vibratome blade with 70% ethanol to remove any residual grease or contaminants. Place the blade in the blade holder and firmly secure it. Adjust the vibratome settings in continuous cutting mode as follows: speed: 0.1; amplitude: 1.6; slice thickness: 350 μm. Cool the cutting chamber to approximately 0°C using an external cooling system.
  *Critical steps:* Incomplete removal of grease or an improperly fixed blade can result in uneven cutting or tissue tearing. Ensure that all grease is removed and that the blade is tightly secured. We recommend performing a vibrocheck according to the manufacturer’s instructions after inserting the blade to verify optimal vibratome performance.
4. Take 0.5 L choline-chloride solution from the refrigerator and place it on ice. Oxygenate the choline-chloride solution for at least 30 min using carbogen (5% CO_2_/95% O_2_) while keeping it on ice. Immediately before starting the slicing procedure, fill the cutting chamber with the pre-cooled and pre-oxygenated choline-chloride solution, and continue oxygenation during the entire cutting procedure to maintain high oxygen levels. Do not place the carbogen tube too close to the blade in order to avoid disturbances in the slicing process.
5. Pre-heat a water bath to 35 °C next to the vibratome. Fifteen minutes prior to perfusion, place the recovery chamber containing approximately 300 mL aCSF in the water bath and oxygenate continuously with carbogen (5% CO_2_/95% O_2_).
  *Critical steps*: Allow the aCSF to equilibrate to 35 °C and verify the temperature with a thermometer before transferring slices. Place the oxygenation tubing in the corners of the recovery chamber to minimize direct contact of acute slices with gas bubbles, which can cause mechanical damage or local pH shifts.
6. Fill the holding chamber with aCSF and allow it to warm to room temperature under continuous oxygenation (5% CO_2_/95% O_2_). Check the temperature before starting the perfusion to ensure that the holding conditions are stable when slices are transferred after recovery.
7. Prepare the perfusion and slicing benches for the perfusion procedure and the preparation of acute slices, ensuring that all instruments and solutions are readily accessible (Figure 1B).
  *Critical steps*: Perform perfusion and slicing in two dedicated workspaces/benches in close proximity to each other. Avoid long distances or unnecessary transfers between steps, as delays in cooling and oxygenation can reduce tissue viability, particularly in aged animals.
8. Prime the butterfly needle with oxygenated choline-chloride solution to remove air and fill a 20 mL syringe with choline-chloride solution directly before perfusion, keeping it on ice until use.
  *Critical steps*: Ensure that the solution is ice-cold and well oxygenated, as inadequate cooling or oxygenation during perfusion can compromise tissue integrity and reduce the quality of subsequent acute slices.

#### Transcardial perfusion (~ 5 min)

1. Mice are anesthetized using an appropriate isoflurane vaporizer system. Animals are exposed to 5% isoflurane vaporized in O_2_ until asphyxia with loss of spontaneous movement, respiratory arrest, and absence of reflexes is reached. To ensure the absence of pain prior to perfusion, assess the absence of pain responses using the intertendinous reflex (toe pinch).
  *Critical steps*: All legal permissions to perform isoflurane euthanasia followed by transcardial perfusion must be obtained before starting experiments. Prepare all instruments and solutions in advance to minimize the duration of anesthesia and avoid *post mortem* delays. Every effort must be made to reduce pain or distress in the animals, and perfusion should only be initiated once asphyxia and the absence of pain is confirmed.
2. Position the mouse on the perfusion table and securely fix the limbs. Ensure continuous exposure to isoflurane via a head mask throughout the surgical procedure.
  *Critical steps*: Although asphyxia has been reached before surgery, continuous isoflurane exposure provides an additional safety margin against any residual nociceptive perception during thoracotomy and perfusion.
3. Remove the skin and rapidly open the abdominal cavity through a midline longitudinal incision. Using scissors and forceps, remove the anterior rib cage by cutting along the ventral axillary line from the inferior to the superior thoracic aperture to fully expose the heart, lungs, and liver.
  *Critical steps*: Ensure that both pleural cavities are opened to create a bilateral pneumothorax. Avoid accidental damage to major vessels (e.g., aorta, vena cava), as uncontrolled bleeding can impair effective perfusion and complicate subsequent procedures.
4. Perform a venting incision in the right atrium or in the liver of the mouse to allow efficient blood drainage from the vascular system during perfusion. Place the butterfly needle in the left ventricle and slowly perfuse the mouse with approximately 12–15 mL of choline-chloride solution at a flow rate of 250 μL/s. A reliable sign of successful perfusion is the liver turning from deep red to a pale color.
  *Critical steps*: Accurate placement of the butterfly needle in the left ventricle (via an apical puncture) is critical. Avoid perforation of the interventricular septum, ventricular wall, or aorta, as this will reduce perfusion efficiency and may damage the heart. Maintain a slow and constant flow rate to preserve tissue integrity and prevent vascular rupture, especially in aged animals. Both right atrial and liver incisions can serve as effective venting sites. We recommend a deep incision of the right liver lobe to avoid additional manipulation of the heart. Carefully remove air from the perfusion system before starting and avoid excessive pressure at any time. Successful perfusion is a critical prerequisite for obtaining high-quality acute brain slices.
5. After perfusion, rapidly decapitate the animal and immediately proceed with the preparation of acute slices. Coordinate with other research groups in advance to share organs when possible, thereby reducing the overall number of animals required for experimental analyses.

#### Tissue extraction and acute slicing procedure (~ 10 min + 1 h recovery)

1. Perform all subsequent steps on a pre-cooled metal block (4 °C) to ensure continuous cooling of the tissue during extraction. Remove the skin and open the skull bilaterally along the skull base from the cerebellum to the olfactory bulb using scissors. Carefully separate the skull plates without penetrating or compressing the underlying brain tissue, with particular attention to the target region (here, the medial prefrontal cortex). Use forceps to remove the skull anteriorly up to the olfactory bulb, thereby fully exposing the dorsal surface of the brain (Figure 1C).
  *Critical steps*: Do not place the metal block in a freezer, as partial freezing of the tissue severely impairs mechanical properties, compromises slice quality, and hampers subsequent electrophysiological recordings. Ensure that the skull is completely opened before attempting to lift it. Incomplete opening often results in sudden release and unwanted movement of the brain. If the skull fractures during removal, carefully lift individual bone fragments from the brain surface with fine forceps. Throughout the procedure, avoid shear stress, indentation, or stretching of the cortical surface, especially in the target region.
2. Using a spatula, gently lift the brain from the skull and transfer it onto the tilted agarose plate in the 35 mm Petri dish, placing its ventral side down on the ~20° agarose slope. With a scalpel, perform a straight transverse cut rostral to the cerebellum to generate a flat posterior surface that will later be attached to the specimen table (included in the standard equipment of the vibratome).
  *Critical steps*: Avoid squeezing, pinching, or compressing the brain with the spatula or forceps, as even brief mechanical compression can cause irreversible tissue damage and reduce slice viability. Avoid tissue warming, since prolonged handling at room temperature negatively affects the quality of acute slices, particularly in aged tissue.
3. Attach the angled cut surface of the brain to the specimen table using a small amount of superglue. Subsequently, position a small agarose block (approximately the same size as the brain) against the ventral side of the brain to provide mechanical support during slicing, and glue the block to the specimen table with superglue (Figures 1D,E).
  *Critical steps*: Ensure that the specimen table is completely dry before applying superglue, as residual moisture reduces adhesion and may cause the tissue to shift during slicing. When positioning the brain on the specimen table, consider that frontal sections will be cut in a dorsal-to-ventral direction. During placement of the agarose support block, take care that superglue does not spread between the brain surface and the agarose, as this will interfere with the cutting plane, create irregular surfaces, and may tear the tissue.
4. In the vibratome cutting chamber, perform frontal sections containing the medial prefrontal cortex with a thickness of 350 μm using a Leica VT1200S vibratome. As soon as the blade reaches the agarose block, gently remove the resulting tissue slices from the brain surface. Keep all acute slices in the cooled, oxygenated solution within the cutting chamber until the slicing procedure is completed.
  *Critical steps*: Ensure that slices do not adhere to the specimen table or the chamber walls. Minimize manual manipulation of slices with tools, since excessive handling increases shear stress and can lead to microlesions that compromise electrophysiological stability.
5. After the slicing procedure is completed, use a wide-diameter glass pipette to transfer slices onto filter membranes in the recovery chamber (aCSF at 35 °C). Avoid up-and-down pipetting or rapid fluid movements at this step. Keep the slices in the recovery solution for 30 min under continuous oxygenation to allow stabilization after cutting.
6. Subsequently, transfer the slices onto filter membranes in the holding chamber (aCSF at room temperature) and allow them to rest for at least 30 min under continuous oxygenation before experimental assessment. This sequence of recovery and holding steps is critical to maximize viability and synaptic stability in acute slices from aged animals.

#### Electrophysiology – plasticity protocol (~ 1.5 h/slice)

1. After recovery, transfer slices to the bath chamber of the electrophysiological setup using a wide-opening glass pipette. Avoid extensive pipetting, as this can impose shear stress on recovered tissue and reduce slice viability. Carefully position the tissue sections so that they lie flat in the recording chamber and do not fold when visualized under the microscope.
  *Critical steps*: During transfer, ensure that slices do not adhere to the inner glass surface or float on top of the liquid column. A shallow pipetting angle and slow release of the slice help to avoid trapping air underneath the tissue. Prevent air bubble formation in the pipette and recording chamber, as bubbles can cause sudden movements or rotation of the slices and lead to mechanical damage.
2. Place a slice hold-down on the tissue section, ensuring that the strings or filaments do not contact the target region selected for electrophysiological recordings. Maintain the maximum feasible distance between the strings and the area of interest. Once the slice holder is placed on the tissue, avoid repositioning it during the recording session, as even subtle movements can introduce shear stress.
3. Continuously perfuse the tissue section with oxygenated and prewarmed (35 °C) aCSF at a flow rate of 2.5 mL/min.
4. Use your patch setup to visualize cells and to amplify and digitize the recordings. We used an LN-Scope (Luigs and Neumann, Germany) equipped with infrared differential interference contrast (IR-DIC) optics and a 40 × water-immersion objective (numerical aperture [NA] 0.8; Olympus) to visually identify neurons. Electrophysiological signals were amplified using a Multiclamp 700B amplifier, digitized with a Digidata 1550B digitizer, and visualized and recorded with the pClamp 11 software package.
  *Critical steps*: At this stage, slice quality should be evaluated by inspecting for dead or swollen cells at the slice surface, macroscopic tissue edema, and evidence of membrane/tissue disruption (see Troubleshooting). If slice quality is insufficient, the slice should be discarded immediately to maintain a time-efficient workflow. Notably, none of the slices included in this study showed extensive tissue damage or overt signs of cellular stress. Therefore, no slices were discarded (for details see Supplementary Table S4).
5. For whole-cell patch-clamp recordings, cells are approached with borosilicate glass pipettes containing internal solution that contains 126 mM K-gluconate, 4 mM KCl, 10 mM HEPES, 4 mM MgATP, 0.3 mM Na_2_GTP, 10 mM phosphocreatine (PO-creatine), and 0.2% (w/v) biocytin (pH 7.25 adjusted with KOH; 285 mOsm/kg). Pipettes have a tip resistance of 3–5 MΩ. After establishing a stable whole-cell configuration, sEPSCs are recorded in voltage-clamp mode at a holding potential of −70 mV.
  *Critical steps:* Continuously monitor series resistance before, and after each recording to detect partial resealing of the membrane patch or deterioration of the seal. Discard recordings if series resistance reaches ≥ 30 MΩ or changes by more than a preset threshold (e.g., > 20%) during the experiment, as this compromises voltage control and data quality. Based on this criterion, one cell was excluded from further analysis of intrinsic membrane properties in aged mice.
6. After characterizing synaptic transmission, intrinsic membrane properties can be further assessed in current-clamp mode. Correct pipette capacitance (2.0 pF) and compensate for series resistance using the automated bridge balance tool in the MultiClamp Commander software. IV-curves are generated by injecting 1 s square current pulses, starting at −100 pA and increasing in 20 pA increments until +600 pA is reached (sweep duration: 2 s). This protocol allows quantification of rheobase, firing frequency, and spike pattern.
  *Critical steps*: The resting membrane potential (RMP) is a useful quality parameter for neuronal functional integrity. Here, an RMP > −50 mV (after *post hoc* correction for the liquid junction potential) was defined as an exclusion criterion. This threshold should be adapted to the targeted cell type and brain region. Notably, no recordings were excluded based on this criterion, supporting high slice quality and preserved neuronal integrity with the present protocol in this study (for details see Supplementary Table S4).
7. After finishing the recording, switch to the membrane oscilloscope mode and gently close the membrane by slowly retracting the glass pipette under continuous visual control. Ideally, the nucleus should remain in the cell to preserve its morphology for *post hoc* analysis. Once the membrane is resealed, remove the pipette from the bath.
  *Critical steps*: After each recording, use a new patch pipette filled with fresh internal solution. Reusing pipettes or internal solution increases the likelihood of tip contamination, poor seals, and unstable recordings.
8. cLTP is induced by forskolin wash-in (10 μM in aCSF, 10 min) in the bath chamber of the electrophysiology setup. To define baseline synaptic transmission prior to forskolin application, sEPSCs and input–output-curves (IV-curves) are recorded in 3 supragranular (layer 2/3) PNs per slice.
9. After completion of baseline recordings in up to 3 PNs per slice (pre), forskolin-containing aCSF is washed into the bathing chamber at a concentration of 10 μM (flow rate: 2.5 mL/min). Following a 10 min forskolin treatment, the drug is washed out again for at least 10 min. Subsequently, electrophysiological recordings are performed in 3 PNs in the same slice (post) to enable an in-slice comparison of excitatory synaptic activity before and after cLTP induction. This paired design reduces variability and improves sensitivity to detect differences in plasticity.
10. When all recordings are completed, transfer the tissue section onto a piece of filter paper using forceps. Once the slice adheres to the filter paper, remove it from the bath chamber and immediately fix the tissue for 1 h in 4% paraformaldehyde (PFA; w/v, in 0.1 M PBS, with 4% (w/v) sucrose), followed by overnight fixation in 2% PFA (w/v, in 0.1 M PBS, with 30% (w/v) sucrose). Maintaining the orientation of the slice is critical for *post hoc* labeling and accurate identification of recorded regions and cell layers. We recommend marking one side of the slice with a small incision before fixation. Perform fixation in a dedicated workspace and strictly follow safety guidelines for handling PFA or other fixatives (e.g., fume hood, appropriate personal protective equipment). Avoid contamination of instruments and surfaces with fixative and thoroughly rinse tools after use.

### *Post hoc* visualization

1. After fixation, wash slices thoroughly in PBS (0.1 M, pH 7.4) to remove residual fixatives. Subsequently, incubate slices for 1 h in blocking solution (PBS containing 10% (v/v) normal goat serum [NGS] and 0.5% (v/v) Triton X-100) to reduce nonspecific staining and to improve penetration of streptavidin and antibodies into the tissue.
2. Next, incubate slices with streptavidin Alexa Fluor 647 (1:1000; #S32357, Invitrogen; in PBS containing 10% (v/v) NGS and 0.1% (v/v) Triton X-100) at 4 °C overnight. Subsequently, apply DAPI nuclear stain (1:1000 in PBS for 20 min; #62248, Thermo Scientific) to visualize cytoarchitecture and to facilitate identification of layer and region. To expand the information gained from the recorded neurons, additional immunostainings (e.g., for cell type markers, synaptic proteins, or glial markers) can be integrated into the staining protocol.
3. After completion of staining, wash slices in PBS to remove unbound fluorophores and antibodies, then carefully transfer them onto glass slides and mount using DAKO anti-fading mounting medium (#S302380-2, Agilent).
  *Critical steps*: During mounting, ensure that the recorded surface of the slice is oriented upwards, minimizing the working distance between the objective and the labeled cells. Use the incision mark introduced before fixation as an orientation reference. Avoid trapping air bubbles under the slice or coverslip, as they distort the optical path and reduce image quality. Proper orientation and bubble-free mounting substantially enhance resolution and signal-to-noise ratio.
4. Acquire images using an appropriate confocal microscope. We used a Leica SP8 laser scanning microscope equipped with a 20 × multi-immersion objective (NA 0.75; Leica) for overview imaging and a 63 × oil-immersion objective (NA 1.4; Leica) for high-resolution structural analysis.

### Quantification and statistics

Electrophysiological data were analyzed using the pClamp 11.3 (Axon instruments) software. sEPSC properties were analyzed using the automated template search tool for event detection. Input resistance was calculated for the injection of −100 pA current at a time frame of 200 ms at the end of the initial hyperpolarization (steady-state hyperpolarization). Resting membrane potential was calculated as the mean baseline value over all sweeps. Liquid junction potential (LJP) was calculated at 14.5 mV (at 35° C) using the LJP calculator of the Clampex 11.3 software. Resting membrane potential was *post hoc* corrected for the LJP (V_m_ = V_rec_ – LJP, with V_rec_ being the recorded voltage).

Data were statistically evaluated using GraphPad Prism 10 (GraphPad Software, USA). Recordings were obtained from three mice per dataset, with two slices per mouse. Within each slice, 2–3 neurons were recorded before forskolin wash-in and an additional 2–3 neurons were recorded after forskolin wash-out (young mice: n_pre_ = 15 and n_post_ = 18 neurons in 6 slices from 3 animals; aged mice: n_pre_ = 17 and n_post_ = 15 neurons in 6 slices from 3 animals, one cell was excluded from intrinsic property analysis in the pre wash-in group in aged mice (n_pre_ = 16) due to the loss of patch-integrity). Neuron-level comparisons of pre versus post conditions across all recordings were performed using the Mann–Whitney test, with individual neurons treated as data points. To address the paired structure within slices, we additionally calculated slice means (average of all neurons recorded per slice for pre and post forskolin application) and compared pre versus post values using the paired Wilcoxon signed-rank test. While the use of the C57BL/6 J inbred background and both standardized housing and recording conditions is expected to reduce between-animal variability, the data retain a hierarchical structure (neurons nested within slices and animals). Therefore, studies with larger inter-individual heterogeneity and/or greater animal numbers may benefit from nested statistical tests or mixed-effects models. However, the use of individual neurons as data points for statistical analysis remains widely established in the field. *p*-values < 0.05 were considered statistically significant (*p < 0.05, **p < 0.01, ***p < 0.001), results that did not yield significant differences are designated ‘ns’.

### Digital illustrations

Confocal images were stored as.tif files, and image brightness and contrast were adjusted. Figures were prepared using the ImageJ software package (https://imagej.net) and Photoshop graphics software (Adobe, San Jose, CA, USA).

## Troubleshooting

- Perfusion is not optimal: If suboptimal perfusion is recognized during the perfusion process (e.g., the liver remains dark red, peripheral vessels remain filled, or blood drainage is insufficient), carefully reposition the butterfly needle within the left ventricle and verify its apical placement. Ensure that the ventricular wall has not been perforated and that perfusion pressure remains moderate, as excessive pressure can rupture vessels and damage brain tissue. Suboptimal perfusion markedly reduces the viability and physiological quality of slice preparations. If sufficient perfusion cannot be achieved despite careful repositioning, consider repurposing the brain, for example by immediate snap-freezing for subsequent molecular biology analyses, rather than proceeding with electrophysiology.
- Tissue is not cut properly and adheres to the brain: If tissue is not cut properly and sections adhere to the remaining brain block, first verify that both the brain and the supporting agarose block are firmly glued to the specimen table and cannot move relative to each other. Confirm that the blade is securely mounted in the blade holder and that slicing parameters (amplitude, speed, and thickness) match those specified in the protocol. When using continuous slicing mode, readjust the front and rear borders of the slicing window so that the blade fully traverses the tissue and slightly enters the agarose block. We recommend continuing the slicing procedure until the vibrating blade clearly reaches the agarose support. Gently remove each tissue section from the slicing area using a suitable tool, minimizing mechanical contact with the cutting edge of the slice to avoid compression or tearing.
- Dead neurons on top of the slices: Dead or degenerating neurons on the slice surface typically show intense membrane contrast, soma shrinkage, blebbing, or ballooning. If many such cells are present, evaluate potential causes systematically. First, confirm that all solutions (choline-chloride solution, aCSF) have the correct composition, pH, osmolarity, and temperature, and that they are adequately oxygenated throughout perfusion, slicing, and recovery. Second, reassess perfusion quality and tissue handling: ensure efficient blood drainage, rapid cooling before brain removal, and minimal delay between decapitation and immersion of the brain in ice-cold, oxygenated solution. Timing is essential in these steps, since any delays from decapitation to recovery of slices lead to reduced tissue quality. Third, avoid excessive mechanical stress: perform all dissections gently, prevent squeezing of the tissue, and minimize shear stress when transferring slices. If these factors are optimized and surface degeneration persists, re-examine the cutting angle and orientation to avoid truncating apical dendrites of PNs and exposing vulnerable cell compartments at the slice surface.
- Unstable patch: If the patch is unstable or breaks shortly after establishing the whole-cell configuration, consider that you may be patching neurons too close to the slice surface, where tissue damage is more pronounced. Surface neurons often exhibit strong contrast and sharp borders, which can be visually attractive but frequently indicate compromised viability. Instead, target neurons located slightly deeper in the slice that display smoother membranes and more homogeneous contrast. Identifying and patching neurons in the vicinity of blood vessels can be advantageous, as intact vascular structures tend to stabilize surrounding tissue during slicing and are often associated with better-preserved cells.
- Neuronal membranes keep closing during the recordings: If neurons repeatedly reseal after break-in (indicated by a rapid increase in pipette resistance to ≥ 30 MΩ or loss of access), a likely cause is that the nucleus shifts toward the pipette tip, promoting closure of the access pathway. To reduce this risk, avoid advancing the pipette too far into the soma during seal formation and break-in. After gaining access, gently applying a small amount of positive pressure can help maintain a stable whole-cell configuration by preventing cytoplasmic components from occluding the pipette tip. However, excessive pressure can disrupt the seal or cause the cell to swell. Therefore, adjust pressure cautiously and monitor series resistance continuously.
- Blurred confocal images: If confocal images appear blurred, lack subcellular resolution, or if labeled cells seem to be located very deep within the tissue, first verify the orientation of the slice on the slide. The surface containing the patched and labeled neurons must face upwards. If slices were inadvertently flipped during mounting, carefully remove the coverslip in PBS and re-mount the slices with the correct orientation, using the incision mark as a reference. Next, confirm that the imaging setup is appropriately configured: use the correct objective (e.g., 63 × oil-immersion for high-resolution imaging), verify the immersion medium, and optimize acquisition parameters such as pinhole size, pixel dwell time, sampling rate, and z-step size. Reducing scanning speed or adjusting averaging can further improve signal-to-noise ratio without excessively increasing photobleaching.
- Truncated apical dendrites: If *post hoc* visualization reveals that neurons have truncated apical dendrites, reassess whether the cutting angle is appropriate for the specific cortical region and dendritic orientation. With an agarose slope angle of approximately 20° for the medial prefrontal cortex, the first slices obtained at the vibratome may be too rostral, and the alignment of apical dendrites can promote truncation. To reduce this risk, discard the most rostral slice and evaluate dendritic preservation in subsequent sections. If truncation persists, fine-tune the angle of the agarose slope and the positioning of the brain on the specimen table to better align the cutting plane with the longitudinal axis of apical dendrites.

## Results

### Slices of young and aged mice present intact cortical lamination and dendritic as well as synaptic morphology

**Figure 2:**
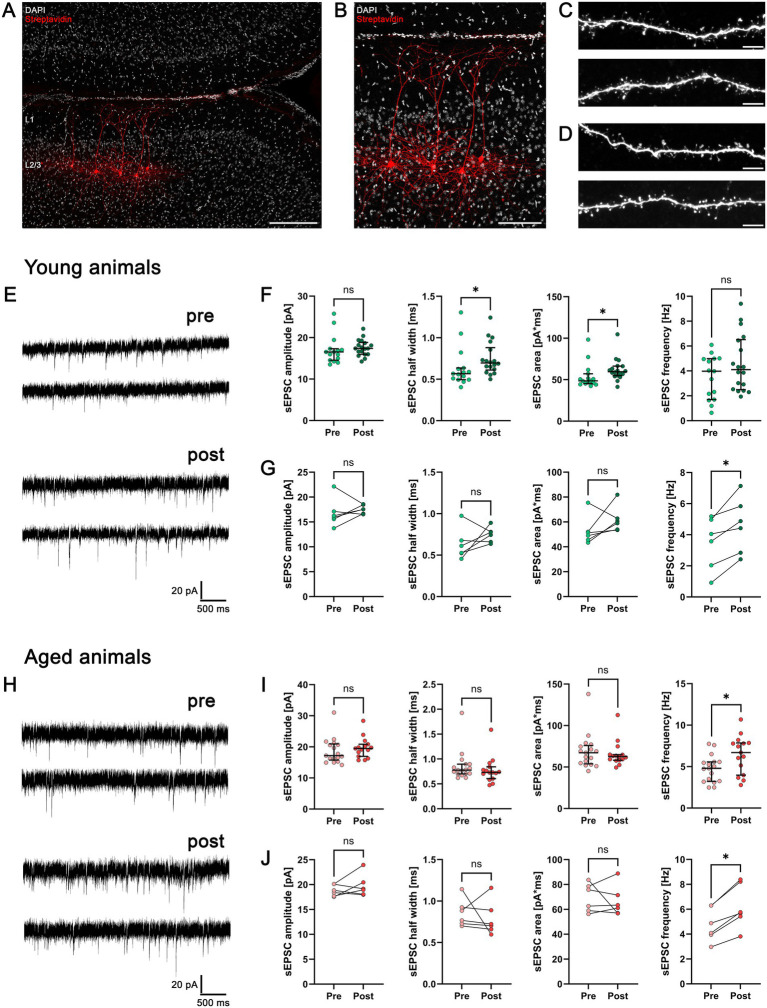
Forskolin-induced cLTP reveals age-specific excitatory synaptic plasticity in pyramidal neurons (PNs) of young and aged animals. **(A)** Representative image of the mPFC stained with DAPI nuclear stain showing an intact cortical lamination with *post hoc* visualized PNs. Layer 1, L1; Layer 2/3, L2/3. Scale bar 200 μm. **(B)** *Post hoc* visualized supragranular (L2/3) PNs with preserved dendritic architecture. Scale bar 100 μm. **(C,D)** Representative images of dendritic segments in young **(C)** and aged mice **(D)**. The upper images show apical dendrites whereas the lower images show basal dendrites. Scale bar 5 μm. **(E,H)** Sample traces of whole-cell patch-clamp recordings of sEPSCs in PNs of young **(E)** and aged mice **(H)**. **(F)** Group data of sEPSC recordings in PNs of young mice before (pre) and after (post) forskolin wash-in (*n*_pre_ = 15 cells, *n*_post_ = 18 cells in three animals; Mann–Whitney test). **(G)** In-slice comparison of sEPSC recordings in young mice before (pre) and after (post) forskolin wash-in (mean of recorded cells pre and post wash-in in *n* = 6 slices of three mice; Wilcoxon matched-pairs signed rank test). **(I)** Group data of sEPSC recordings in PNs of aged mice before (pre) and after (post) forskolin wash-in (*n*_pre_ = 17 cells, *n*_post_ = 15 cells in three animals; Mann–Whitney test). **(J)** In-slice comparison of sEPSC recordings in aged mice before (pre) and after (post) forskolin wash-in (mean of recorded cells pre and post wash-in in *n* = 6 slices of three mice; Wilcoxon matched-pairs signed rank test). Individual data points are indicated by dots. Values represent median ± interquartile range (*, *p* < 0.05; ns, non-significant difference). Source data are provided in Supplementary Table S1 (young) and Supplementary Table S2 (aged).

We used acute slice preparations of adult female mice at two age points (young: 7–10 weeks; aged: 9–11 months) and performed whole-cell patch-clamp recordings from supragranular (layer 2/3) PNs in the medial prefrontal cortex (mPFC). Consecutive *post hoc* visualization of biocytin-filled, patched neurons in combination with DAPI staining of nuclei revealed an intact cortical lamination in the recorded slices from both age groups (Figures 2A,B). Basal and apical dendrites of these neurons were not truncated by the slicing procedure and exhibited a highly branched dendritic architecture.

High-resolution imaging of dendritic segments was used to assess dendritic spines as the structural correlate of excitatory postsynaptic compartments. In neurons from both young (Figure 2C) and aged (Figure 2D) mice, dendritic spine structure and the diversity of spine morphologies appeared preserved, without obvious qualitative differences between age groups. Together, these observations indicate that our protocol maintains the structural integrity of supragranular mPFC neurons, even in aged mice, and thereby provides a suitable framework for the assessment of synaptic function and plasticity in structurally intact neurons.

### Supragranular (layer 2/3) PNs in slices of young and aged mice show age-specific expression of excitatory synaptic plasticity upon forskolin-induced cLTP

The ability of neurons to undergo plastic changes in response to appropriate stimuli is a prerequisite for learning and higher cognitive function. To test whether supragranular (layer 2/3) PNs in the mPFC of young and aged mice differ in their expression of excitatory synaptic plasticity, we applied a chemical LTP (cLTP) protocol based on forskolin wash-in (10 μM for 10 min). Whole-cell patch-clamp recordings were used to assess spontaneous excitatory neurotransmission in up to three neurons per slice before forskolin application to define baseline activity (Figures 2E,H; pre). At least 10 min after forskolin wash-out, sEPSCs were recorded from neurons in the same slice to quantify forskolin-induced changes in excitatory transmission (Figures 2E,H; post).

In young adult animals, forskolin treatment led to a significant strengthening of excitatory neurotransmission. When comparing neurons across all animals, sEPSC half-width and area were significantly increased after forskolin exposure (*p* = 0.02, half-width; *p* = 0.01, area; Mann–Whitney test; Figure 2F). Slice-wise pre-post paired comparisons of the mean values of recorded neurons (in-slice control) further revealed a significant increase in sEPSC frequency upon cLTP induction (*p* = 0.03; Wilcoxon matched-pairs signed rank test; Figure 2G).

In aged mice, forskolin application resulted in a significant increase in sEPSC frequency when neurons from all animals were compared between pre and post conditions, whereas sEPSC amplitude, half-width, and area remained unchanged (*p* = 0.02, frequency; Mann–Whitney test; Figure 2I). This frequency-specific effect was corroborated by pre-post paired comparisons of neurons recorded in the same slice (*p* = 0.03; Wilcoxon matched-pairs signed rank test; Figure 2J). Together, these data indicate that supragranular (layer 2/3) PNs in the mPFC of both young and aged animals retain the capacity to adjust excitatory neurotransmission in response to forskolin-induced cLTP. However, the pattern of synaptic changes differs with age: in young animals, plasticity is expressed as both an increase in sEPSC frequency and altered sEPSC kinetics (half-width and area), whereas in aged animals it is predominantly reflected by an increase in event frequency.

**Figure 3:**
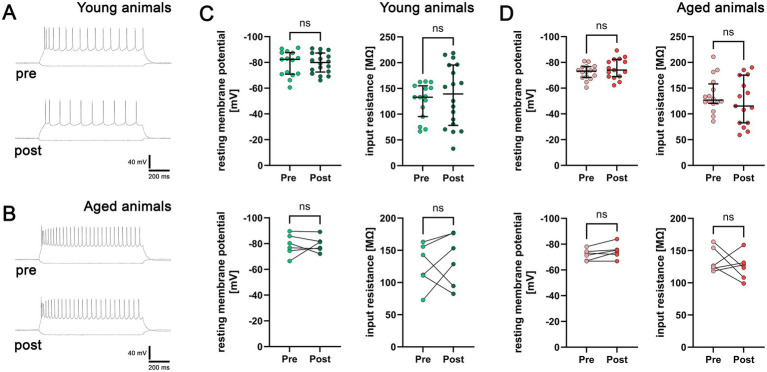
Passive membrane properties of PNs remain unchanged after forskolin wash-in. **(A,B)** Sample traces of input–output curves in young **(A)** and aged mice **(B)**. **(C)** Group data and in-slice comparison of resting membrane potentials and input resistances in young mice (group data: *n*_pre_ = 15 cells, *n*_post_ = 18 cells in three animals; Mann–Whitney test. In-slice comparison: mean of recorded cells pre and post wash-in in *n* = 6 slices of three mice; Wilcoxon matched-pairs signed rank test). **(D)** Group data and in-slice comparison of resting membrane potentials and input resistances in aged mice (group data: *n*_pre_ = 16 cells, *n*_post_ = 15 cells in three animals, one cell was excluded from intrinsic property analysis in the pre wash-in group due to the loss of patch-integrity; Mann–Whitney test. In-slice comparison: mean of recorded cells pre and post wash-in in *n* = 6 slices of three mice; Wilcoxon matched-pairs signed rank test). Individual data points are indicated by dots. Values represent median ± interquartile range (ns, non-significant difference). Source data are provided in Supplementary Table S3.

To determine whether these synaptic changes are accompanied by alterations in passive membrane properties, we recorded input–output curves (Figures 3A,B) and measured resting membrane potential and input resistance of patched neurons before and after forskolin exposure. None of these parameters showed significant differences between pre and post conditions in slices from young (Figure 3C) or aged animals (Figure 3D). We therefore conclude that forskolin-induced cLTP of excitatory neurotransmission in supragranular mPFC neurons occurs without detectable changes in passive membrane properties in either age group.

## Discussion

In this study, we used an standardized choline-chloride perfusion protocol to obtain acute slices of the mPFC from young and aged mice. Using this protocol, we were able to maintain the structural integrity of neurons in aged tissue, including dendritic spines as the structural correlate of excitatory synapses. Whole-cell patch-clamp recordings revealed a forskolin-induced strengthening of excitatory neurotransmission in supragranular (layer 2/3) PNs in both age groups. This effect was reflected by an increase in sEPSC frequency, while intrinsic membrane properties remained unchanged. At the same time, we identified age-dependent differences in sEPSC half-width and area, indicating that the pattern of plasticity expression differs between young and aged animals. These findings are consistent with a growing literature indicating that ageing can alter both the magnitude and the expression profile of synaptic plasticity (Radulescu et al., 2025). Of note, we used female mice to establish this experimental framework across age, but the present design does not permit conclusions regarding sex differences. Multiple studies have reported sex-dependent plasticity phenotypes across brain regions, including the involvement of different signaling pathways (Brokling et al., 2022; Heo et al., 2025; Hyer et al., 2018; Tiwari et al., 2025). In addition, several pathways central to plasticity, such as GluN2B-associated signaling, BDNF/TrkB-dependent mechanisms, and neuro-immune signaling, have been implicated in both ageing-related and sex-related variability of synaptic function (Faraji et al., 2022; Imiruaye et al., 2025; Le et al., 2024; Muller et al., 2025; Nikoletopoulou et al., 2017). Future studies could therefore employ this protocol to systematically test how biological sex interacts with ageing to shape synaptic transmission and plasticity. Together, these findings establish an experimental framework for assessing synaptic transmission and plasticity in the mouse brain across ages and open new opportunities to investigate age-related changes in neuronal network function, including those relevant to neurodegenerative diseases.

*Ex vivo* acute slice preparations of brain tissue are a well-established tool in neuroscience, as they allow functional assessment of living neurons under controlled conditions. The success of such preparations critically depends on rapid processing and on the ionic composition and temperature of cutting and recovery solutions. Tissue from young adult mice is comparatively robust and – with sufficient experience – can be prepared reproducibly. In contrast, tissue from aged animals is more vulnerable, showing increased sensitivity to hypoxia and osmotic stress (Wang et al., 2018; Djurisic, 2020; Kolenicova et al., 2020) and enhanced stiffness that complicates the cutting procedure (Chuang et al., 2023). In addition, Na^+^-induced depolarization during slicing can trigger excitotoxicity and neuronal degeneration (Choi, 1987; Raley-Susman et al., 2001), which is more pronounced in aged brains. To mitigate these age-related vulnerabilities, we performed transcardial perfusion with ice-cold choline-chloride solution prior to slicing. Perfusion with well-oxygenated, ice-cold protective media, such as choline-chloride or NMDG-aCSF, has been shown to improve tissue viability by minimizing ischemia, reducing oxidative stress, and facilitating cutting with less tissue tearing (Ting et al., 2018; Kékesi and Buskila, 2020). Notably, we did not directly quantify oxidative stress and did not apply additional antioxidant measures during preparation and recording in either young or aged brains. While antioxidant supplementation may improve slice quality in specific experimental settings, no overt limitations in slice quality were observed here. Conversely, the absence of exogenous antioxidants may facilitate interrogation of endogenous age-dependent differences in oxidative stress-related processes.

Consistent with previous reports, we identified perfusion with a protective solution prior to slicing emerged as a critical determinant of tissue quality. Using the proposed protocol, we reliably obtained viable mPFC slices from aged mice that displayed intact cortical lamination and preserved dendritic architecture, thereby enabling the analysis of age-dependent structure–function relationships. Although we did not perform a systematic comparison of protective solutions in the present study, previous work indicates that cutting and recovery paradigms can markedly influence edema, neuronal preservation, and recording yield in adult and aging tissue (Ting et al., 2018; Pan et al., 2015). In particular, comparative studies suggest that NMDG-based protective recovery can improve neuronal preservation and recording yield relative to traditional sucrose-based protective cutting approaches. Since the choline-chloride workflow presented here shows high protective capacity in both young and naturally aged mice, we regard this protocol as a robust option that can be implemented with reduced preparation time and straightforward handling in routine acute-slice pipelines. We reliably obtained aged mPFC slices with preserved lamination and dendritic architecture and could quantify forskolin-induced synaptic potentiation, supporting its suitability for plasticity assays across age. In principle, this protocol can be adapted for use in other brain regions, which is relevant for investigating vulnerable circuits in ageing and neurodegeneration, including the hippocampus in mouse models of Alzheimer’s disease. Such adaptations typically require region- and model-specific adjustments, particularly in tissue handling, slicing procedure (e.g., Bischofberger et al., 2006), and electrophysiological settings (including inclusion/exclusion criteria), as it was previously established in other protocols (Baccini et al., 2021; Halfmann et al., 2023; Huang and Uusisaari, 2013; Willett et al., 2019). Notably, in the present study we did not require age-specific parameter changes between young and aged animals, supporting the suitability of this workflow for standardized plasticity assays across age. We anticipate that the same workflow can be applied to accelerated ageing models. Depending on the pathology, including potentially infectious proteinopathies and prion-like disorders, additional safety procedures may be required based on a project-specific risk assessment.

Neurodegenerative diseases, such as Alzheimer’s disease, are frequently accompanied by impairments in the brain’s capacity to respond appropriately to plasticity-inducing stimuli, resulting in memory deficits, disorientation, and cognitive decline (Shankar et al., 2008; Chapman et al., 1999). Understanding how synaptic plasticity is expressed and modulated during aging is therefore of considerable interest. Whole-cell patch-clamp recordings provide direct access to synaptic events at the single-neuron level. Here, we used a forskolin-based protocol to induce chemical LTP (cLTP) for probing excitatory synaptic plasticity in structurally intact supragranular PNs (c.f., Figures 2A–D). It is interesting to speculate that alternative protocols, such as electrical (pathway-specific) stimulation, might be even more reliant on the structural integrity of the tissue and synaptic compartments. Neurons from both young and aged adult mice showed a forskolin-induced increase in sEPSC frequency, indicating that excitatory synapses in the mPFC retain a basic capacity for activity-independent, cAMP-driven potentiation across age. Notably, in young adult mice, forskolin treatment additionally increased sEPSC half-width and area, whereas these parameters remained unchanged in aged mice. This pattern suggests age-dependent differences in the mechanisms by which plasticity is implemented. Forskolin is known to activate adenylate cyclase and increase intracellular cAMP levels, thereby enhancing presynaptic release probability by increasing the readily releasable pool of vesicles (Kaneko and Takahashi, 2004; Sakaba and Neher, 2001). On the postsynaptic side, cAMP-dependent signaling can promote phosphorylation of AMPA receptor subunits and their insertion into the postsynaptic density, resulting in synaptic strengthening (Roche et al., 1996; Esteban et al., 2003). In addition, cAMP-dependent cascades can engage mTOR-dependent protein synthesis of plasticity-related proteins, supporting more persistent forms of synaptic modification (Maity et al., 2020; Fuchsberger et al., 2025). Within this framework, it is plausible that the observed increase in sEPSC frequency primarily reflects presynaptic changes that are preserved in both age groups, whereas the additional changes in sEPSC half-width and area in young mice may indicate a more pronounced postsynaptic contribution. These kinetic and integrative parameters are often sensitive to alterations in postsynaptic receptor kinetics, receptor availability, and receptor recruitment or clustering (Yang et al., 2025; Stincic and Frerking, 2015). Although our data do not directly dissect pre- and postsynaptic mechanisms, they are consistent with the notion that postsynaptic components of cAMP-dependent plasticity may be selectively attenuated or altered in aged supragranular PNs.

Despite the advantages of the present approach, several limitations of acute slice preparations and *ex vivo* plasticity paradigms must be considered. Even when tissue viability and lamination are preserved, the slicing procedure inevitably causes mechanical trauma and acute glial cell responses, which may influence subsequent plasticity-related signaling (Taubenfeld et al., 2002). Although glial cells and the microvasculature are affected by the preparation, glia–neuron-interactions can be assessed in viable sections over short time windows, particularly when suitable reporter mouse lines or cell type-specific labeling strategies are available. Moreover, long-range cortical and thalamo-cortical projections are severed during slicing, leading to an artificial isolation of local circuits and a loss of physiological input patterns (Stepanyants et al., 2009; Capone et al., 2019). These network-level alterations should be considered when interpreting *ex vivo* circuit-level findings in both young and aged brains, particularly when extrapolating to the *in vivo* situation. Nonetheless, acute slice preparations offer a unique opportunity to combine high-resolution structural analysis with precise electrophysiological measurements at the single-cell level. In the context of aging and neurodegeneration, the improved workflow presented here enables reproducible investigations of synaptic transmission and plasticity in structurally preserved neurons of the aged neocortex and thus provides a valuable platform for dissecting cellular mechanisms that contribute to age-related cognitive decline and disease-associated network dysfunction.

## Data Availability

The raw data supporting the conclusions of this article will be made available by the authors, without undue reservation.

## References

[ref1] Ando M. Oku N. Takeda A. (2010). Zinc-mediated attenuation of hippocampal mossy fiber long-term potentiation induced by forskolin. Neurochem. Int. 57, 608–614. doi: 10.1016/j.neuint.2010.07.010, 20674642

[ref2] Baccini G. Brandt S. Wulff P. (2021). Preparation of acute slices from dorsal hippocampus for whole-cell recording and neuronal reconstruction in the dentate gyrus of adult mice. J. Vis. Exp. doi: 10.3791/61980 33871459

[ref3] Bischofberger J. Engel D. Li L. Geiger J. R. Jonas P. (2006). Patch-clamp recording from mossy fiber terminals in hippocampal slices. Nat. Protoc. 1, 2075–2081. doi: 10.1038/nprot.2006.312, 17487197

[ref4] Brokling J. Brunne B. Rune G. M. (2022). Sex-dependent responsiveness of hippocampal neurons to sex neurosteroids: a role of arc/Arg3.1. J. Neuroendocrinol. 34:e13090. doi: 10.1111/jne.13090 35081672

[ref5] Campagnola L. Seeman S. C. Chartrand T. Kim L. Hoggarth A. Gamlin C. . (2022). Local connectivity and synaptic dynamics in mouse and human neocortex. Science 375:eabj5861. doi: 10.1126/science.abj5861 35271334 PMC9970277

[ref6] Capone C. Rebollo B. Munoz A. Illa X. Del Giudice P. Sanchez-Vives M. V. . (2019). Slow waves in cortical slices: how spontaneous activity is shaped by laminar structure. Cereb. Cortex 29, 319–335. doi: 10.1093/cercor/bhx326, 29190336

[ref7] Chapman P. F. White G. L. Jones M. W. Cooper-Blacketer D. Marshall V. J. Irizarry M. . (1999). Impaired synaptic plasticity and learning in aged amyloid precursor protein transgenic mice. Nat. Neurosci. 2, 271–276. doi: 10.1038/6374, 10195221

[ref8] Choi D. W. (1987). Ionic dependence of glutamate neurotoxicity. J. Neurosci. 7, 369–379. doi: 10.1523/JNEUROSCI.07-02-00369 2880938 PMC6568907

[ref9] Chuang Y. C. Alcantara A. Fabris G. Abderezaei J. Lu T. A. Melendez-Vasquez C. V. . (2023). Myelination dictates axonal viscoelasticity. Eur. J. Neurosci. 57, 1225–1240. doi: 10.1111/ejn.15954 36878871 PMC12882786

[ref10] Djurisic M. (2020). Minimizing hypoxia in hippocampal slices from adult and aging mice. J. Vis. Exp. doi: 10.3791/61377 32716375

[ref11] Esteban J. A. Shi S. H. Wilson C. Nuriya M. Huganir R. L. Malinow R. (2003). PKA phosphorylation of AMPA receptor subunits controls synaptic trafficking underlying plasticity. Nat. Neurosci. 6, 136–143. doi: 10.1038/nn997, 12536214

[ref12] Faraji J. Lotfi H. Moharrerie A. Jafari S. Y. Soltanpour N. Tamannaiee R. . (2022). Regional differences in BDNF expression and behavior as a function of sex and enrichment type: oxytocin matters. Cereb. Cortex 32, 2985–2999. doi: 10.1093/cercor/bhab395, 35059698

[ref13] Fuchsberger T. Stockwell I. Woods M. Brzosko Z. Greger I. H. Paulsen O. (2025). Dopamine increases protein synthesis in hippocampal neurons enabling dopamine-dependent LTP. eLife 13:RP100822. doi: 10.7554/eLife.100822 40063079 PMC11893101

[ref14] Halfmann C. Ruland T. Muller F. Jehasse K. Kampa B. M. (2023). Electrophysiological properties of layer 2/3 pyramidal neurons in the primary visual cortex of a retinitis pigmentosa mouse model (rd10). Front. Cell. Neurosci. 17:1258773. doi: 10.3389/fncel.2023.1258773, 37780205 PMC10540630

[ref15] Heo S. Zhang S. Mun D. G. Pandey A. Bygrave A. M. Huganir R. L. (2025). Sex and experience dependent regulation of synaptic protein turnover. bioRxiv. doi: 10.1101/2025.11.24.690161, 41394749 PMC12697534

[ref16] Huang S. Uusisaari M. Y. (2013). Physiological temperature during brain slicing enhances the quality of acute slice preparations. Front. Cell. Neurosci. 7:48. doi: 10.3389/fncel.2013.00048, 23630465 PMC3632751

[ref17] Hyer M. M. Phillips L. L. Neigh G. N. (2018). Sex differences in synaptic plasticity: hormones and beyond. Front. Mol. Neurosci. 11:266. doi: 10.3389/fnmol.2018.00266, 30108482 PMC6079238

[ref18] Imiruaye O. E. Perez I. G. Carson B. C. Crouzet C. Garcia J. Han D. . (2025). Spatiotemporal differential regulation of extrasynaptic GluN2B receptor subunits and PSA-NCAM in brain aging and Alzheimer's disease. Front. Neurosci. 19:1649625. doi: 10.3389/fnins.2025.1649625, 40948811 PMC12426952

[ref19] Kaneko M. Takahashi T. (2004). Presynaptic mechanism underlying cAMP-dependent synaptic potentiation. J. Neurosci. 24, 5202–5208. doi: 10.1523/jneurosci.0999-04.2004, 15175390 PMC6729197

[ref20] Kékesi O. Buskila Y. (2020). Method for prolonged incubation of brain slices. Bio Protoc. 10:e3683. doi: 10.21769/BioProtoc.3683 PMC7842590 33659354

[ref21] Kolenicova D. Tureckova J. Pukajova B. Harantova L. Kriska J. Kirdajova D. . (2020). High potassium exposure reveals the altered ability of astrocytes to regulate their volume in the aged hippocampus of GFAP/EGFP mice. Neurobiol. Aging 86, 162–181. doi: 10.1016/j.neurobiolaging.2019.10.009, 31757575

[ref22] Le A. A. Lauterborn J. C. Jia Y. Cox C. D. Lynch G. Gall C. M. (2024). Metabotropic NMDAR signaling contributes to sex differences in synaptic plasticity and episodic memory. J. Neurosci. 44:e0438242024. doi: 10.1523/JNEUROSCI.0438-24.2024, 39424366 PMC11638816

[ref23] Ma Y. Wan J. Hao S. Chen Q. Y. Zhuo M. (2024). Recruitment of cortical silent responses by forskolin in the anterior cingulate cortex of adult mice. Mol. Pain 20:17448069241258110. doi: 10.1177/17448069241258110, 38744422 PMC11119478

[ref24] Maity S. Chandanathil M. Millis R. M. Connor S. A. (2020). Norepinephrine stabilizes translation-dependent, homosynaptic long-term potentiation through mechanisms requiring the cAMP sensor Epac, mTOR and MAPK. Eur. J. Neurosci. 52, 3679–3688. doi: 10.1111/ejn.14735, 32275785

[ref25] McIntyre D. C. Hutcheon B. Schwabe K. Poulter M. O. (2002). Divergent GABA(a) receptor-mediated synaptic transmission in genetically seizure-prone and seizure-resistant rats. J. Neurosci. 22, 9922–9931. doi: 10.1523/JNEUROSCI.22-22-09922.2002, 12427849 PMC6757834

[ref26] Morris R. G. Anderson E. Lynch G. S. Baudry M. (1986). Selective impairment of learning and blockade of long-term potentiation by an N-methyl-D-aspartate receptor antagonist, AP5. Nature 319, 774–776. doi: 10.1038/319774a0, 2869411

[ref27] Muller L. Di Benedetto S. Muller V. (2025). Influence of biological sex on neuroinflammatory dynamics in the aging brain. Front. Aging Neurosci. 17:1670175. doi: 10.3389/fnagi.2025.1670175, 40951920 PMC12426254

[ref28] Nikoletopoulou V. Sidiropoulou K. Kallergi E. Dalezios Y. Tavernarakis N. (2017). Modulation of autophagy by BDNF underlies synaptic plasticity. Cell Metab. 26, 230–242 e5. doi: 10.1016/j.cmet.2017.06.005 28683289

[ref29] Otmakhov N. Khibnik L. Otmakhova N. Carpenter S. Riahi S. Asrican B. . (2004). Forskolin-induced LTP in the CA1 hippocampal region is NMDA receptor dependent. J. Neurophysiol. 91, 1955–1962. doi: 10.1152/jn.00941.2003, 14702333

[ref30] Pan G. Li Y. Geng H. Y. Yang J. M. Li K. X. Li X. M. (2015). Preserving GABAergic interneurons in acute brain slices of mice using the N-methyl-D-glucamine-based artificial cerebrospinal fluid method. Neurosci. Bull. 31, 265–270. doi: 10.1007/s12264-014-1497-1 25648546 PMC5562650

[ref31] Perumal M. B. Sah P. (2022). A protocol to investigate cellular and circuit mechanisms generating sharp wave ripple oscillations in rodent basolateral amygdala using ex vivo slices. STAR Protoc 3:101085. doi: 10.1016/j.xpro.2021.101085, 35072114 PMC8761775

[ref32] Popescu A. T. Pare D. (2011). Synaptic interactions underlying synchronized inhibition in the basal amygdala: evidence for existence of two types of projection cells. J. Neurophysiol. 105, 687–696. doi: 10.1152/jn.00732.2010, 21084688 PMC3357011

[ref33] Radulescu C. I. Pilch K. S. Wang X. Gibbs F. Barnes S. J. (2025). Turning back time: aging plasticity and its rejuvenation. Curr. Opin. Neurobiol. 94:103097. doi: 10.1016/j.conb.2025.103097, 40829306

[ref34] Raley-Susman K. M. Kass I. S. Cottrell J. E. Newman R. B. Chambers G. Wang J. (2001). Sodium influx blockade and hypoxic damage to CA1 pyramidal neurons in rat hippocampal slices. J. Neurophysiol. 86, 2715–2726. doi: 10.1152/jn.2001.86.6.2715, 11731531

[ref35] Roche K. W. O'Brien R. J. Mammen A. L. Bernhardt J. Huganir R. L. (1996). Characterization of multiple phosphorylation sites on the AMPA receptor GluR1 subunit. Neuron 16, 1179–1188. doi: 10.1016/s0896-6273(00)80144-0, 8663994

[ref36] Sakaba T. Neher E. (2001). Preferential potentiation of fast-releasing synaptic vesicles by cAMP at the calyx of held. Proc. Natl. Acad. Sci. USA 98, 331–336. doi: 10.1073/pnas.98.1.331 11134533 PMC14590

[ref37] Saunders A. Granger A. J. Sabatini B. L. (2015). Corelease of acetylcholine and GABA from cholinergic forebrain neurons. eLife 4:e06412. doi: 10.7554/eLife.06412 25723967 PMC4371381

[ref38] Shankar G. M. Li S. Mehta T. H. Garcia-Munoz A. Shepardson N. E. Smith I. . (2008). Amyloid-beta protein dimers isolated directly from Alzheimer's brains impair synaptic plasticity and memory. Nat. Med. 14, 837–842. doi: 10.1038/nm1782 18568035 PMC2772133

[ref39] Stepanyants A. Martinez L. M. Ferecsko A. S. Kisvarday Z. F. (2009). The fractions of short- and long-range connections in the visual cortex. Proc. Natl. Acad. Sci. USA 106, 3555–3560. doi: 10.1073/pnas.0810390106, 19221032 PMC2651285

[ref40] Stincic T. L. Frerking M. E. (2015). Different AMPA receptor subtypes mediate the distinct kinetic components of a biphasic EPSC in hippocampal interneurons. Front. Synaptic Neurosci. 7:7. doi: 10.3389/fnsyn.2015.00007, 26042027 PMC4434957

[ref41] Taubenfeld S. M. Stevens K. A. Pollonini G. Ruggiero J. Alberini C. M. (2002). Profound molecular changes following hippocampal slice preparation: loss of AMPA receptor subunits and uncoupled mRNA/protein expression. J. Neurochem. 81, 1348–1360. doi: 10.1046/j.1471-4159.2002.00936.x, 12068082

[ref42] Ting J. T. Daigle T. L. Chen Q. Feng G. (2014). Acute brain slice methods for adult and aging animals: application of targeted patch clamp analysis and optogenetics. Methods Mol. Biol. 1183, 221–242. doi: 10.1007/978-1-4939-1096-0_14, 25023312 PMC4219416

[ref43] Ting J. T. Lee B. R. Chong P. Soler-Llavina G. Cobbs C. Koch C. . (2018). Preparation of acute brain slices using an optimized N-methyl-D-glucamine protective recovery method. J. Vis. Exp. 53825. doi: 10.3791/53825 29553547 PMC5931343

[ref44] Tiwari M. N. Paik L. da Silva Fiorin F. Chung M. K. (2025). Sex differences of synaptic plasticity and microglial remodeling in the dorsal hippocampus following trigeminal nerve injury in mice. Neurobiol. Dis. 215:107097. doi: 10.1016/j.nbd.2025.107097 40935162 PMC12494171

[ref45] Turrigiano G. G. Leslie K. R. Desai N. S. Rutherford L. C. Nelson S. B. (1998). Activity-dependent scaling of quantal amplitude in neocortical neurons. Nature 391, 892–896. doi: 10.1038/36103, 9495341

[ref46] Wang F. Yang Y. J. Yang N. Chen X. J. Huang N. X. Zhang J. . (2018). Enhancing oligodendrocyte myelination rescues synaptic loss and improves functional recovery after chronic hypoxia. Neuron 99, 689–701 e5. doi: 10.1016/j.neuron.2018.07.017, 30078577 PMC6170028

[ref47] Wen J. Liang Z. Li C. Pang H. Jiang L. Li J. . (2025). Motor-cognitive aging: the role of motor cortex and its pathways. NeuroImage 320:121472. doi: 10.1016/j.neuroimage.2025.121472 40972830 PMC13060929

[ref48] Whitebirch A. C. LaFrancois J. J. Jain S. Leary P. Santoro B. Siegelbaum S. A. . (2022). Enhanced excitability of the hippocampal CA2 region and its contribution to seizure activity in a mouse model of temporal lobe epilepsy. Neuron 110, 3121–3138 e8. doi: 10.1016/j.neuron.2022.07.020, 35987207 PMC9547935

[ref49] Willett J. A. Cao J. Dorris D. M. Johnson A. G. Ginnari L. A. Meitzen J. (2019). Electrophysiological properties of medium spiny neuron subtypes in the caudate-putamen of prepubertal male and female Drd1a-tdTomato line 6 BAC transgenic mice. eNeuro 6:ENEURO.0016-19.2019. doi: 10.1523/eneuro.0016-19.2019, 30899778 PMC6426437

[ref50] Yang Y. Wong M. H. Huang X. Chiu D. N. Liu Y. Z. Prabakaran V. . (2025). Distinct transmission sites within a synapse for strengthening and homeostasis. Sci. Adv. 11:eads5750. doi: 10.1126/sciadv.ads5750, 40215296 PMC11988405

